# Antiferromagnetic and ferromagnetic spintronics and the role of in-chain and inter-chain interaction on spin transport in the Heisenberg ferromagnet

**DOI:** 10.1038/s41598-021-99813-9

**Published:** 2021-10-14

**Authors:** L. S. Lima

**Affiliations:** Department of Physics, Federal Center for Technological Education of Minas Gerais, Belo Horizonte, MG 30510-000 Brazil

**Keywords:** Magnetic properties and materials, Spintronics

## Abstract

Spin-transport and current-induced torques in ferromagnet heterostructures given by a ferromagnetic domain wall are investigated. Furthermore, the continuum spin conductivity is studied in a frustrated spin system given by the Heisenberg model with ferromagnetic in-chain interaction $J_1<0$ between nearest neighbors and antiferromagnetic next-nearest-neighbor in-chain interaction $J_2>0$ with aim to investigate the effect of the phase diagram of the critical ion single anisotropy $D_c$ as a function of $J_2$ on conductivity. We consider the model with the moderate strength of the frustrating parameter such that in-chain spin-spin correlations that are predominantly ferromagnetic. In addition, we consider two inter-chain couplings $J_{\perp ,y}$ and $J_{\perp ,z}$, corresponding to the two axes perpendicular to chain where ferromagnetic as well as antiferromagnetic interactions are taken into account.

## Introduction

From the end of 80 decade up to now, the spintronics has witnessed a variety of spin related phenomena such as spin transfer torque, tunneling magnetoresistance^1^ and so on. The spintronics demands the spin transport study, where the spin current plays a central role in order of spintronics phenomena to occur in magnetic materials and also in various other materials including semiconductors and oxides^2,3^. So, the spin current can be used to control the magnetization via spin-transfer-torque and spin-orbit-torque in several magnetic (and also nonmagnetic) materials^4–9^.

On the other hand, the frustrated antiferromagnetism has been also a rich topic nowadays since to very intriguingly phenomena like topological phase transitions that may occur in this class of systems. The frustration leads to an importance of quantum effects because of the classical order is suppressed and novel phases may occur and to govern the physics at low-energy. Moreover, these systems can exhibit a nematic ground state induced by a spontaneous symmetry breaking induced by terms such as frustrating interactions in the Heisenberg model^10^. This nematic ordering can occur when spin fluctuations are taken at some axis without any direction being chosen^11^. In a general way, the frustration is present in materials as LiVCuO_4_, LiVCu_2_O_4_, LiZrCuO_4_ that are adequately described by ferromagnetic nearest-neighbor interactions $J_1$ and antiferromagnetic next-nearest-neighbor interactions $J_2$^12–30^. All of these materials are of spin-1/2. For spin-1, there are a small number of materials as example the material NiCl_2_4SC(NH_2_)_2_ which is a quasi-one-dimensional antiferromagnet with easy-plane anisotropy dominating the exchange interaction^31–33^.

The first generation of spintronic devices are based on spin transport, that utilises the magneto-transport being invented in 2001^34^. The injection efficiency depends on the spin polarisation of the ferromagnet and the spin scattering at the ferromagnetic/non-magnetic interface, where it is also important to eliminate any other effects, namely a stray field from a ferromagnet, that distorts the estimation of the injection efficiency.

The aim of this paper is to analyze the effect of scattering among electrons with a ferromagnetic wall domain on spin transport by electrons and to analyse the spin transport by ions on the lattice in a two-dimensional frustrated spin lattice model with the aim to analyse the effect of quantum phase transition (QPT) on longitudinal spin conductivity. Additionally, we analyze the Meissner effect for the ideal spin transport or superconductor obtained for many other two-dimensional frustrated spin systems. The plan of this paper is the following. In “Spin polarized current” section, we discuss the spin polarized current. In “Meissner effect for the spin supercurrent” section, we discuss the Meissner mechanism for the spin super-current. In “Spin transport in quantum frustrated spin-1/2 ferromagnet” section, we discuss the spin transport in afrustrated lattice model such as the Heisenberg ferromagnetic model with in-chain and inter-chain interactions with the aim to analyse the effect of the parameters of coupling in the neighboring of the QPT, on spin conductivity. In the last “Conclusions” section, we present our conclusions and final remarks.

## Spin polarized current

**Figure 1:**
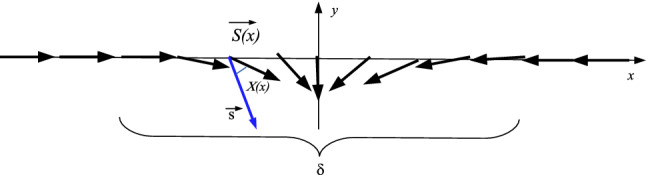
A schematic view of interaction among electron with the spins of the ferromagnetic domain wall of width $\delta$. $\chi (x)$ is the phase angle among electron spin with spin of the ferromagnetic wall.

The spin transport by electrons in a system can be expressed by a spin current in the form $\mathcal {J}_S=-\hbar /2e\left( \mathcal {J}_{\uparrow }-\mathcal {J}_{\downarrow }\right)$, where *e* is the electron charge. In the same way the charge current $\mathcal {J}_c$ is given by $\mathcal {J}_c=\mathcal {J}_{\uparrow }+\mathcal {J}_{\downarrow }$. Both currents obey to a diffusion equation given by

1 $$\begin{aligned} \nabla ^2({\mu }_{\uparrow }-{\mu }_{\downarrow }) =\frac{1}{\Gamma }({\mu }_{\uparrow }-{\mu }_{\downarrow }) \end{aligned}$$

where ${\mu }_{\uparrow ,\downarrow }$ is the magnetic momentum of each electron and $\Gamma =\sqrt{D\tau }$ is the diffusion coefficient, being *D* the diffusion constant and $\tau$ the spin flip time^35,36^. Here, we consider the model described by the Hamiltonian

2 $$\begin{aligned} \mathcal {H}=- \frac{\hbar ^2}{2m}\nabla ^{2} \psi (\mathbf {r})+\mathfrak {V}(\mathbf {r})\psi (\mathbf {r}) +J\mathbf {s}\cdot \mathbf {S}(\mathbf {r})\psi (\mathbf {r}). \end{aligned}$$

where *J* denotes the exchange integral and $\mathfrak {V}(\mathbf {r})$ is the potential (nonmagnetic) of the lattice. The last term in the Equation above $V(\mathbf {r})=J\mathbf {s}\cdot \mathbf {S}(\mathbf {r}')$ provides the interaction between electron with the ferromagnetic domain wall. We consider a homogeneous magnetic domain wall with a collinear magnetization. The interaction among the electrons spins with the spins of the ferromagnetic wall is represented in Fig. 1, where the potential of interaction among the electron spins with the spins of the wall domain is given by $V(\mathbf {r})=J\mathbf {s}\cdot \mathbf {S}(\mathbf {r})$. The the aim here is to verify the influence of this on spin wave function of the electrons. The transmission of electron through a domain wall was discussed in Refs.^37,38^. The purpose here is to use the Born’s expansion for $f_{\uparrow ,\downarrow }( \mathbf {k} ,\mathbf {k'})$ to calculate the effect of interaction electron spin-domain wall on spin wave function of the electrons of the current $\mathcal {J}_c$. The wave function of the electron at large distance from the wall domain is given by

3 $$\begin{aligned} |\psi (\mathbf {r})\rangle =\left( \begin{array}{c} \phi _{\uparrow }(\mathbf {r}) \\ \phi _{\downarrow }(\mathbf {r}) \\ \end{array} \right) , \end{aligned}$$

where $\psi _{in} (\mathbf {r})= \psi (-\infty )$ is the wave function of the electron after the scattering with the wall. Consequently, far from domain wall, we have $\psi _{out} (\mathbf {r})$ given by

4 $$\begin{aligned} |\psi _{\uparrow }(\mathbf {k},\mathbf {r})\rangle =e^{i\mathbf {k}\cdot \mathbf {r}}\left( \begin{array}{c} 1 \\ 0 \\ \end{array} \right) +\frac{e^{i\mathbf {k}\cdot \mathbf {r}}}{r}f_{\uparrow }(\mathbf {k},\mathbf {k'})\left( \begin{array}{c} 0 \\ 1 \\ \end{array} \right) \nonumber \\ |\psi _{\downarrow }(\mathbf {k},\mathbf {r})\rangle =e^{i\mathbf {k}\cdot \mathbf {r}}\left( \begin{array}{c} 0 \\ 1 \\ \end{array} \right) +\frac{e^{i\mathbf {k}\cdot \mathbf {r}}}{r}f_{\downarrow }(\mathbf {k},\mathbf {k'})\left( \begin{array}{c} 1 \\ 0 \\ \end{array} \right) \end{aligned}$$

where $|\psi _{\uparrow ,\downarrow }(\mathbf {k},\mathbf {r})\rangle =\mathbf {S}|\psi _{\uparrow ,\downarrow }(\mathbf {k},\mathbf {r})\rangle$ and $\mathbf {S}$ is the scattering matrix.

5 $$\begin{aligned} f_{\uparrow ,\downarrow }(\mathbf {k},\mathbf {k'})=-\frac{\mu _B}{2\pi \hbar ^{2}} \langle \psi _{\uparrow ,\downarrow }(\mathbf {k},\mathbf {r})|T|\psi _{\uparrow ,\downarrow } (\mathbf {k'},\mathbf {r'})\rangle , \end{aligned}$$

where $\mu _B$ is the Bohr’s magneton and *T* is given by the Lipmann-Schwinger’s equation

6 $$\begin{aligned} T=V+V\frac{1}{\omega -\mathcal {H}_{0}+i0^{+}} \end{aligned}$$

and

7 $$\begin{aligned} \mathcal {H}_{0}= & {} - \frac{\hbar ^2}{2m}\nabla ^{2} +\mathfrak {V}(\mathbf {r}), \end{aligned}$$

8 $$\begin{aligned} V(\mathbf {r})= & {} J\mathbf {s}\cdot \mathbf {S}(\mathbf {r}), \end{aligned}$$

where we consider $J=1$. Thus

9 $$\begin{aligned} f_{\uparrow ,\downarrow }(\mathbf {k},\mathbf {k'}) =\sum _{n=0}^{\infty }f_{\uparrow ,\downarrow }^{(n)}(\mathbf {k},\mathbf {k'}), \end{aligned}$$

where *n* is the times number that *V* enters in the equation above,

10 $$\begin{aligned} f_{\uparrow ,\downarrow }^{(1)}(\mathbf {k},\mathbf {k'}) =-\frac{\mu _B}{2\pi ^2\hbar }\langle \psi _{\uparrow ,\downarrow } (\mathbf {k},\mathbf {r})|V|\psi _{\uparrow ,\downarrow }(\mathbf {k'},\mathbf {r'})\rangle \end{aligned}$$

and the potential *V*(*x*) (${r}=x\hat{i}$) is given by

11 $$\begin{aligned} V(x')=-\frac{2J_{sd}}{g\mu _{B}}\mathbf {s}\cdot \langle \mathbf {S}(x')\rangle , \end{aligned}$$

where $x'$ corresponds the region inside of the ferromagnetic wall domain. Consequently, we have

12 $$\begin{aligned} f_{\uparrow ,\downarrow }^{(1)}(\mathbf {k},\mathbf {k'})=-\frac{2}{e}A(\mathbf {k},\mathbf {k'}) \end{aligned}$$

where the *A*(*k*) coefficient is given by

13 $$\begin{aligned} A(k)=\int _{\frac{-\delta }{2}}^{+\frac{\delta }{2}}S\cos (\arctan (e^{-\delta x'}))\sin (kx')dx'. \end{aligned}$$

**Figure 2:**
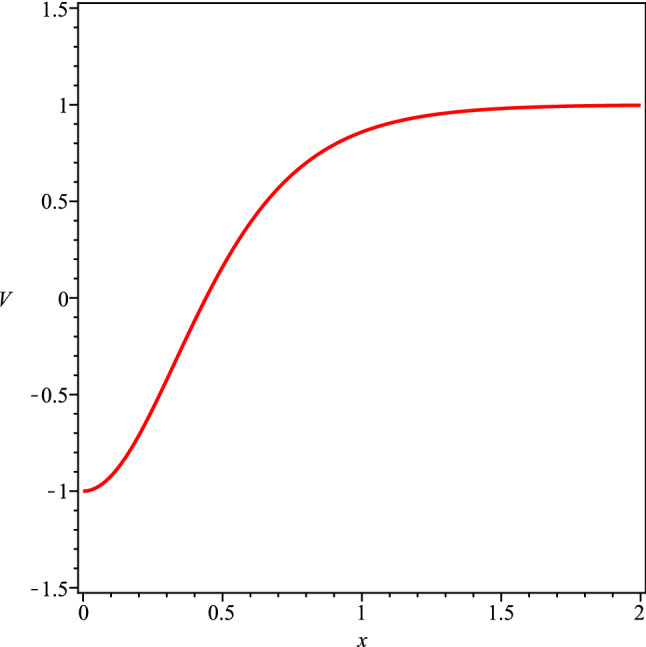
Behavior of the potential of interaction between the electron with the ferromagnetic domain wall, *V*(*x*), where the width of the wall is $\delta$.

The integral above was solved approximately as

14 $$\begin{aligned}A(k)\simeq -\frac{1}{4(k^2+\delta ^2)}\bigg [S\sqrt{2} \bigg (e^{\frac{\delta ^2}{2}}k\cos \bigg (\frac{\delta k}{2}\bigg )\delta ^3 +e^{\frac{\delta ^2}{2}}k^3\cos \bigg (\frac{\delta k}{2}\bigg ) \delta -4e^{\frac{\delta ^2}{2}}k\cos \bigg (\frac{\delta k}{2}\bigg )\delta \nonumber \\&\quad +e^{\frac{\delta ^2}{2}}\sin \bigg (\frac{\delta k}{2}\bigg ) \delta ^4-e^{\frac{\delta ^2}{2}}k\sin \bigg (\frac{\delta k}{2}\bigg )\delta ^2k^2 +2e^{\frac{\delta ^2}{2}}\sin \bigg (\frac{\delta k}{2}\bigg ) \delta ^2-2e^{\frac{\delta ^2}{2}}k\sin \bigg (\frac{\delta k}{2}\bigg )k^2\nonumber \\&\quad +e^{\frac{-\delta ^2}{2}}k\cos \bigg (\frac{\delta k}{2}\bigg ) \delta ^4+e^{-\frac{\delta ^2}{2}}k^3\cos \bigg (\frac{\delta k}{2}\bigg )\delta +e^{\frac{-\delta ^2}{2}}k\cos \bigg (\frac{\delta k}{2}\bigg ) \delta ^4+e^{-\frac{\delta ^2}{2}}k\sin \bigg (\frac{\delta k}{2}\bigg )\delta ^4\nonumber \\&\quad +e^{\frac{-\delta ^2}{2}}\sin \bigg (\frac{\delta k}{2}\bigg ) \delta ^2k^2+2e^{-\frac{\delta ^2}{2}}k\sin \bigg (\frac{\delta k}{2}\bigg )\delta ^2 -2e^{\frac{-\delta ^2}{2}}\sin \bigg (\frac{\delta k}{2}\bigg )k^2\bigg )\bigg ], \end{aligned}$$

where $\delta$ is the width of the wall. The potential of interaction among electron spin with the spins of the domain wall *V*(*x*) has the form $V(x)=J_{sd}S\cos (4\arctan (e^{-\delta x}))$. The shape of the potential is displayed in Fig. 2. We consider the expansion of the Eq. (9) up to first order. An analysis considering terms of superior order will generate a large quantity of terms in the Eq. (14) and should not generate any change in the scattering. We obtain a very complicated expression for the wave function of the electron after the scattering with the ferromagnetic wall domain however, in a combination of two polarization states. The presence of the coefficient $f(\mathbf {k},\mathbf {k'})$ in the second term making the control of the state of polarization of each electron after the scattering with the domain wall a very difficult problem.

*The electron Hamiltonian interacting with the ferromagnetic domain wall can be written as*^39,40^

15 $$\begin{aligned} \mathcal {H}=\mathcal {H}_{0}+\mathcal {H}_{sw}+\mathcal {H}_{w}, \end{aligned}$$

*where* $\mathcal {H}_{0}$ *is the Hamiltonian of the free electron,* $\mathcal {H}_{sw}$ *is the electron-domain-wall Hamiltonian and* $\mathcal {H}_{w}$ *is the Hamiltonian of the wall domain.*

16 $$\begin{aligned} \mathcal {H}_{0}= & {} -t\sum _{\langle ij\rangle } \left( c_{i\uparrow }^{\dag }c_{j\downarrow } + h.c.\right) +\lambda \sum _{i,j}n_{i\uparrow }n_{j\downarrow }, \end{aligned}$$

17 $$\begin{aligned} \mathcal {H}_{sw}= & {} V\sum _{i,j}S_{i}\cdot s_{j}, \end{aligned}$$

18 $$\begin{aligned} \mathcal {H}_{w}= & {} J\sum _{i,j}S_{i}\cdot S_{j}. \end{aligned}$$

*V* *is given by* Eq. (11).

*Making the transformation of the spin operators*

19 $$\begin{aligned} S_{i}^{+}= & {} \sqrt{2S}A_{i}^{\dag },\quad s_{i}^{+}=\sqrt{2S}a_{i}^{\dag }, \end{aligned}$$

20 $$\begin{aligned} S_{i}^{-}= & {} \sqrt{2S}A_{i},\quad s_{i}^{-}=\sqrt{2S}a_{i}, \end{aligned}$$

21 $$\begin{aligned} S_{i}^{z}= & {} S-A_{i}^{\dag }A_i,\quad s_{i}^{z}=s-a_{i}^{\dag }a_i, \end{aligned}$$

we have for the Hamiltonian $\mathcal {H}_{sw}$

22 $$\begin{aligned} \mathcal {H}_{sw}=2V\sqrt{Ss}\sum _{i,j}\left( A_{i}^{\dag }a_{j}+h.c.\right) +\mathcal {H}' \end{aligned}$$

where $\mathcal {H}'$ contains terms of four or more operators $a_i$ and $A_i$. The contribution of the interaction between each electron with the domain wall on the electric current operator $\mathcal {J}_{sw}$ is given by

23 $$\begin{aligned} \mathcal {J}_{sw}\simeq 2V\sqrt{Ss}\sum _{i,j}\left( A_{i}^{\dag }a_{j}-h.c.\right) . \end{aligned}$$

**Figure 3:**
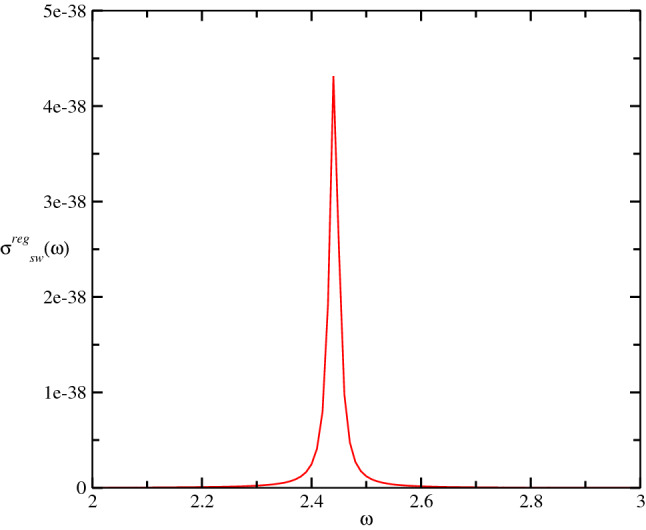
Behavior of the contribution of the interaction between electron with the domain-wall, $\sigma ^{reg}_{sw}(\omega )$ in the temperature $T=0.1J$. The very small value of this contribution is due to interaction of only one electron with the ferromagnetic three-dimensional wall domain. In the electric current we have a flow of *N* electrons by seconds.

We use the Matsubara’s Green function method at finite temperature^39,40^ to determine the contribution of the interaction of the electrons with the wall domain for the regular part of the electric conductivity or continuum conductivity, $\sigma ^{reg}(\omega )$, given by

24 $$\begin{aligned} \sigma _{sw}^{reg}(\omega )=\frac{4V^2sS}{\omega }\sum _{\mathbf {k}} \frac{\sin ^{2}k_x}{\omega _{\mathbf {k}}\mathcal {W}_{\mathbf {k}}} \left[ f_{\mathbf {k}}(N_{\mathbf {k}}+1)\delta (\omega -\omega _{\mathbf {k}} -\mathcal {W}_{\mathbf {k}})+N_{\mathbf {k}}(1-f_{\mathbf {k}}) \delta (\omega +\omega _{\mathbf {k}}+\mathcal {W}_{\mathbf {k}})\right] , \end{aligned}$$

where $f_{k}=\langle n_{i\uparrow }\rangle =\langle n_{i\downarrow }\rangle =(e^{\beta \omega _{\mathbf {k}}}+1)^{-1}$ is the fermion occupation number and $N_{\mathbf {k}}=(e^{\beta \mathcal {W}_{\mathbf {k}}}-1)^{-1}$ is the boson occupation number associated with the spin waves of the wall domain and $\beta =1/T$. We have that in the low energy limit

25 $$\begin{aligned} \omega _{k}= & {} v|{\mathbf {k}}| , \end{aligned}$$

26 $$\begin{aligned} \mathcal {W}_{\mathbf {k}}= & {} \frac{J}{3}\left( \cos k_x,+\cos k_y + \cos k_z\right) \end{aligned}$$

where *v* is the Fermi’s velocity. In the Fig. 3, we present the behavior of the contribution of the interaction among electrons with the domain wall, $\sigma _{sw}^{reg}(\omega )$. How the electric resistance is the inverse of the electric conductivity, the inverse of $\sigma _{sw}^{reg}(\omega )$ provides the information about the electric resistance generated by the ferromagnetic domain wall. Our results show a peak of resonance in the contribution of the spin electron-ferromagnetic wall domain at range $\omega \simeq 2.5 J$, which indicates a peak in the electric conductivity at this range of $\omega$.

## Meissner effect for the spin supercurrent

It is a fact well known that an example in the nature of local spontaneous breaking of gauge symmetry is the superconductivity. How the charge conductivity is a response to a time-dependent electric field given by Ohm’s law, $\mathcal {J}(x,t)=\sigma {E}(x,t)$, in a similar way, we have that the spin current flows in response to a magnetic-field gradient following the Fick’s laws for $\nabla {B}(x,t)$ as $\langle \mathcal {J}(x,t)\rangle =\sigma \nabla {B}(x,t)$. Hence, so as for electric superconductors, we should have here $\sigma \rightarrow \infty$ for the case of a spin superconductor, where in a finite system with *N* sites we must have a finite number of spins. As in general, the spin conductivity cannot be infinite consequently, the gradient of the external magnetic field $\nabla {B}(x,t)$ must be zero inside of the a spin superconductor as in the electron superconductivity. So, if ${B}$ is zero in the beginning, it must be zero inside the superconductor even if we apply a gradient of an external magnetic field outside the superconductor. This means that the applied external magnetic field must not depend on *x* inside the superconductor. Consequently, the spins of a spin superconductor should generate a current that screens the external gradient of the magnetic field.

The action of the system becomes invariant under the gauge transformation

27 $$\begin{aligned}A_{\mu }(x)\rightarrow A_{\mu }(x)+\partial _{\mu }\Lambda (x), \end{aligned}$$

28 $$\begin{aligned}\quad \psi (x)\rightarrow \psi (x)e^{iq\Lambda (x)/\hbar }. \end{aligned}$$

We introduce the Goldstone boson field $\phi (x)$ that has the property $\phi (x)=\phi (x)+\Lambda (x)$ and

29 $$\begin{aligned} \psi (x)=e^{iq\phi (x)/\hbar }\psi (x). \end{aligned}$$

where *q* is the charge. Thus, the magnons in the Heisenberg model must be described by a charged scalar field so as the cooper pairs in the electric superconductor where the Lagrangian describing the interaction between this scalar field with the gradient of the electromagnetic field being given by

30 $$\begin{aligned} \mathcal {L}=-\int \frac{1}{4}F_{\mu \nu }F^{\mu \nu }+L_m[A_{\mu }-\partial _{\mu }\phi ], \end{aligned}$$

being $L_m$ a not well known functional.

The Proca’s equation is given by

31 $$\begin{aligned} \partial _{\mu }F^{\mu \nu }+\Delta ^2A^{\nu }=0 \end{aligned}$$

with

32 $$\begin{aligned} (\Box +\Delta ^2)A^{\nu }=0. \end{aligned}$$

Thus, the scalar field $\psi$ produces a mass for the photons following the Higg’s mechanism where the scalar field that describes the spin waves plays the role of a Higg’s field developing a antiferromagnetic vacuum expectation value. So, the photon must acquire a mass $\Delta$ inside the spin superconductor like in the electric superconductor with the wave equation becoming the Klein-Gordon equation for the quantity $\mathfrak {B}$ defined by $\mathfrak {B}=\nabla {B}(x,t)$ given by the massive Klein-Gordon equation

33 $$\begin{aligned} (\Box +\Delta ^2)\mathfrak {B}=0. \end{aligned}$$

Thus, if we apply a gradient of an external magnetic field, the solution of equation above will become $\nabla ^2\mathfrak {B}=0$ for $x<0$ and $(\nabla ^2-\Delta ^2)\mathfrak {B}=0$ for $x>0$. The solution for $x>0$ gives a penetration length given by $l=1/\Delta$, being the inverse of the mass that the photon acquires inside of the spin superconductor.

## Spin transport in quantum frustrated spin-1/2 ferromagnet

*We discuss the role of the interchain coupling on spin transport in coupled frustrated spin-1/2 magnets with a ferromagnet NN in-chain coupling* $J_1<0$ *and an AFM NNN in-chain coupling* $J_2>0$, *where the chains are aligned along the* *x* *axis, and they are coupled along the* *y* *and* *z* *axes by* $J_{\perp ,y}$ *and* $J_{\perp ,z}$, *respectively. Thus the model is given by*

34 $$\begin{aligned} \mathcal {H}=J_1\sum _{\langle i,j\rangle ,x}S_i\cdot S_j+J_2\sum _{\left[ i,j\right] ,x}S_i\cdot S_j+J_{\bot ,y}\sum _{\langle i,j\rangle ,y}S_i\cdot S_j +J_{\bot ,z}\sum _{\langle i,j\rangle ,y}S_i\cdot S_j, \end{aligned}$$

where $\langle i,j\rangle$, *x*, *y*, *z* labels NN bonds along the corresponding axis, and [*i*, *j*], *x* labels NNN bonds along the chain. Furthermore, we consider $J_1<0$ and $J_2\le 0$, whereas no sign restrictions are valid for $J_{\perp ,y}$ and $J_{\perp ,z}$.

In the linear response theory, the spin conductivity is the response to an actual frequency-dependent gradient of magnetic field is given by^41–43^

35 $$\begin{aligned}  \langle \mathcal {J}_{\beta }(\mathbf {k},\omega )\rangle & =\sum _{\gamma }\sigma _{\beta \gamma }(\mathbf {k},\omega )ik_{\beta } B_{\gamma }(\mathbf {k},\omega ),\nonumber \\ \sigma _{\beta \gamma } & =\hbox {Re}(\sigma _{\beta \gamma }) +i\hbox {Im}(\sigma _{\beta \gamma })\nonumber \\ \sigma ^{reg}(\omega ) & =\frac{\hbox {Im} \{\mathcal {G}({\mathbf {k}}=0,\omega )\}}{\omega }, \end{aligned}$$

where we have introduced the spin-flip part of the exchange interaction along the *x* direction. The Eq. (35) exhibits the desired structure $\langle \mathcal {J}\rangle =\sigma \nabla B^z$, where the formula for the spin conductivity is defined as the linear spin-current response to a uniform, $\mathbf {k}=0$, frequency-dependent gradient of the magnetic field^44^. In general, the $\mathbf {k}=0$ conductivity at $T=0$ may be written as $\hbox {Re}\left( \sigma _{\beta \gamma }(\omega )\right) =D_S\delta (\omega )+\sigma ^{reg}(\omega )$, where $D_S$ is the Drude’s weight. Therefore, beyond the $\mathbf {k}=0$ the relation between the “twist conductivity” and the response to an inhomogeneous magnetic field is not clear.

The Green’s function at zero temperature is defined as^42^

36 $$\begin{aligned} \mathcal {G}(t)\equiv -\frac{i}{N}\langle 0|\mathbf {T}\mathcal {J}_x({\mathbf {k}},t),\mathcal {J}_x(-{\mathbf {k}},0)|0\rangle . \end{aligned}$$

where ${\mathbf {T}}$ is the time ordering operator. The current-response function $\mathcal {G}({\mathbf {k}},\omega )$ at finite temperature is given by

37 $$\begin{aligned} \mathcal {G}({\mathbf {k}},\omega )=\frac{i}{ N}\int _{0}^{\infty }dte^{i\omega t}\langle 0| [\mathcal {J}_x({\mathbf {k}},t),\mathcal {J}_x(-{\mathbf {k}},0)]|0\rangle . \end{aligned}$$

$\mathcal {G}({\mathbf {k}}=0,\omega \rightarrow 0)$ is the susceptibility or retarded Green’s function^42,43^.

The operator for spin current from site *j* to site $j+x$ is defined by^44–48^

38 $$\begin{aligned} \mathcal {J}_x=i\frac{(J_1+J_{\perp ,y}+J_{\perp ,z})}{2} \sum _j(S_{j}^{+}S_{j+x}^{-}-S_{j}^{-}S_{j+x}^{+}) +\frac{iJ_2}{2}\sum _j(S_{j}^{+}S_{j+2x}^{-}-S_{j}^{-}S_{j+2x}^{+}) \end{aligned}$$

where $j+x$ is the nearest-neighbor site of the site *j* in the positive *x* direction. Furthermore, the spin-current operator can be expressed as $\mathcal {J}=-i\left[ \mathcal {X},\mathcal {H}\right]$, where the generating operator $\mathcal {X}$ is $\mathcal {X}\equiv \sum _jx_jS_j^z$, where $x_j$ is the *x*-coordinate of lattice site *j*, $\mathcal {J}_x(j)\equiv \mathcal {J}_{x\rightarrow j+x}$ and $\mathcal {J}_x\equiv \sum _{j}\mathcal {J}_x(j)$.

We find the spin current operator in terms of boson operators $\alpha$ and $\beta$ given by

39 $$\begin{aligned} \mathcal {J}_x=t^{2} \sum _{\mathbf {k}}\left[ \frac{\sin k_{x} (J_1+J_{\perp ,y}+J_{\perp ,z})+J_2\sin (2k_{x})}{\omega _{\mathbf {k}}}\right] \left( \alpha _{\mathbf {k}}+\beta _{\mathbf {k}}\right) \left( \alpha _{\mathbf {k}}^{\dag } +\beta _{\mathbf {k}}^{\dag }\right) , \end{aligned}$$

where the higher-order terms in Eq. (39) involves terms of four or more boson operators and have been discarded. We find the Green’s function given by

40 $$\begin{aligned} \mathcal {G}({\mathbf {k}},\omega )=t^2(J_1+J_{\perp ,y}+J_{\perp ,z})^2 \sum _{\mathbf {k},\mathbf {k}'}\frac{\sin k_{x}'}{\omega _{\mathbf {k}'}} \frac{\sin k_{x}}{\omega _{\mathbf {k}}}\mathfrak {g}_{\mathbf {kk'}}(\omega )+t^2J_2^2 \sum _{\mathbf {k},\mathbf {k}'}\frac{\sin (2k_{x}')}{\omega _{\mathbf {k}'}} \frac{\sin (2k_{x})}{\omega _{\mathbf {k}}}\mathfrak {g}_{\mathbf {kk'}}(\omega ) \end{aligned}$$

where $\mathfrak {g}_{\mathbf {kk'}}(\omega )$ is obtained by applying the Wick’s theorem following of the Fourier transform after performing a tedious calculation. The Eq. (40) corresponds to the lowest approximation (noninteracting magnetic excitations) which replaces the magnon propagators by the free propagators $\mathcal {G}^0$ and hence, it is valid only in the mean-field approach. Thus, the Green’s function for $\psi$ is $\langle \alpha (t)\alpha ^{\dag }(0)\rangle \rightarrow \mathcal {G}_0(t)$ and $\langle \beta ^{\dag }(t)\beta (0)\rangle \rightarrow \tilde{\mathcal {G}}_0(t)$, where $\mathcal {G}_0$ is the bare propagator.

41 $$\begin{aligned} \mathcal {G}(\mathbf {k},\omega )=\frac{1}{\pi ^2}\int _{0}^{2\pi }d \omega _1\mathcal {G}_0(\mathbf {k},\omega _1)\tilde{\mathcal {G}}_0(\mathbf {k},\omega -\omega _1), \end{aligned}$$

and

42 $$\begin{aligned} \tilde{\mathcal {G}}_0(\mathbf {k},\omega )=-\frac{1}{\omega +\omega _{\mathbf {k}}-i0^{+}},\quad {\mathcal {G}}_0(\mathbf {k},\omega )=\frac{1}{\omega -\omega _{\mathbf {k}}+i0^{+}}. \end{aligned}$$

Furthermore, we employ the formula^42^

43 $$\begin{aligned} \frac{1}{2\pi }\int \mathcal {G}_0(\omega ) d\omega \rightarrow T\sum _{m}\mathcal {G}_0(\omega \rightarrow i\omega _m), \end{aligned}$$

We obtain the retarded Green’s function and $\sigma ^{reg}(\omega )$, using the SU(3) Schwinger boson theory and applying the Gree-Kubo formula we obtain the continuum conductivity given by

44 $$\begin{aligned} \sigma ^{reg}(\omega )=t^2\sum _{\mathbf {k}} \frac{\left[ \sin k_{x}(J_1+J_{\perp ,y}+J_{\perp ,z})+J_2\sin (2k_{x})\right] ^2}{\omega _{\mathbf {k}}^3}\delta (\omega -\omega _{\mathbf {k}}), \end{aligned}$$

We find the spin conductivity using the SU(3) Schwinger boson theory given as a second-rank tensor, being different from results in literature obtained using the Dyson-Maleev representation which is given by a scalar or zero-order tensor^42,43^. This difference implies in a different response of the spin current to the gradient of the external magnetic field $\nabla \mathbf {B}$.

We can improve SU(3) Schwinger boson mean-field formalism including the fluctuations around the mean-field result^49^. We can consider the phase fluctuations $\phi _{ij}$ around the mean-field results for *A* as $A_{ij}=\tilde{A}e^{i\phi _{ij}}$ taking into account in the action for the Hamiltonian Eq. (34) which is invariant under the gauge transformation: $b_{ij}\rightarrow b_{ij}+(\phi _i-\phi _j)$, $b_j\rightarrow b_je^{i\phi _j}$.

**Figure 4:**
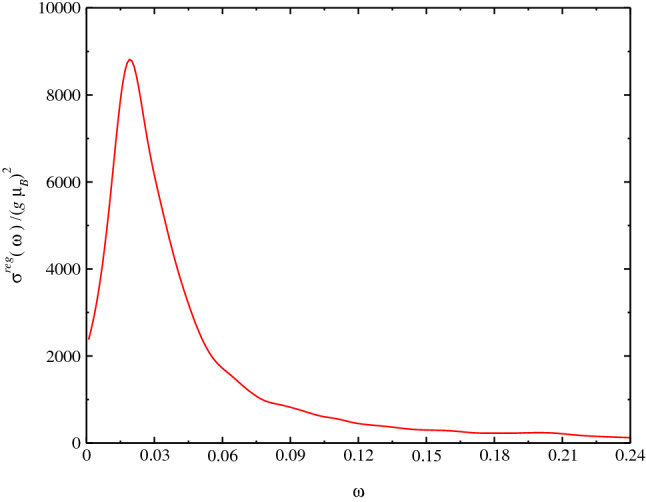
Plot of $\sigma ^{reg}(\omega )$ at $T=0$ using SU(3) Schwinger boson theory). We perform the calculations for values $J_1=-1.0$, $J_{\perp ,y}=0.1$, $J_{\perp ,z}=0.0$ and $J_2=0.2$. The $J_2$ value is near to the phase transition $0.251\le J_{2C}\le 0.252$. The factor $(g\mu _B)^2$ should be put in case of comparison to experimental data.

**Figure 5:**
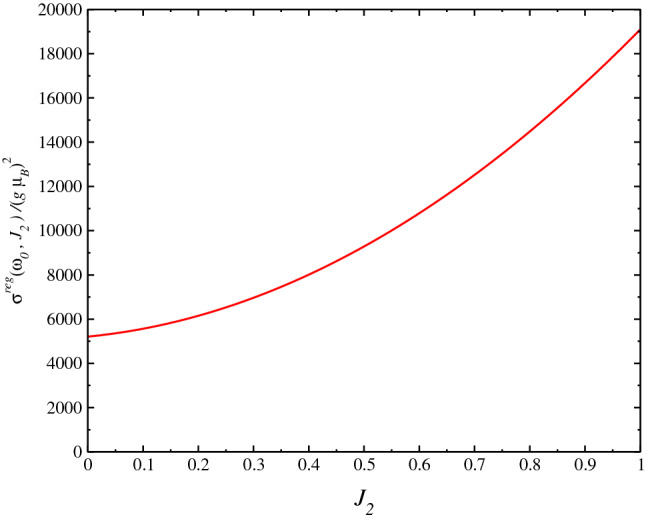
Plot of $\sigma ^{reg}(\omega =\omega _0)/(g\mu _B)^2$ as a function of $J_2$ for $J_1=-1.0$ and $J_{\perp ,y}=0.1$, $J_{\perp ,z}=0.0$. We perform the calculations for a $\omega$ value near to the peak of the spin conductivity $\omega =\omega _p\approx 0.03$. The factor $(g\mu _B)^2$ should be put in case of comparison to experimental data.

**Figure 6:**
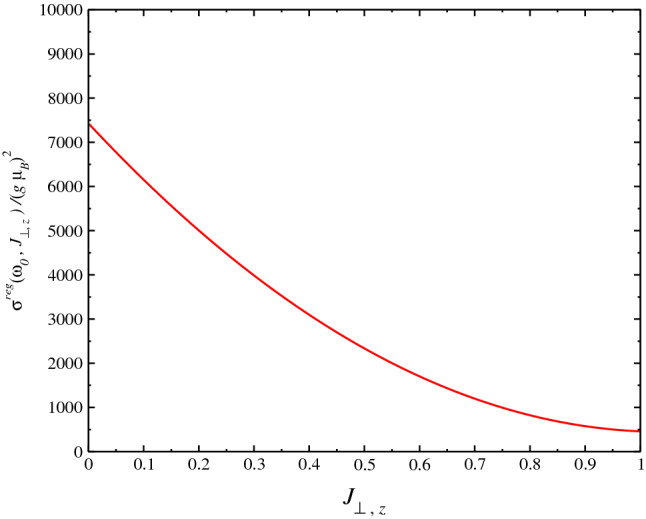
Plot of $\sigma ^{reg}(\omega )$ as a function of $J_z$ for $J_1=-1.0$ and $J_2=0.2$. We perform the calculations for a $\omega$ value near to the peak of the spin conductivity $\omega =\omega _p\approx 0.03$. The factor $(g\mu _B)^2$ should be put in case of comparison to experimental data.

In Fig. 4, we obtain $\sigma ^{reg}(\omega )$ at $T=0$ using the SU(3) Schwinger boson formalism. We perform the calculations for the following values of parameters: $J_1=-1.0$, $J_y=0.1$, $J_z=0.0$ and $J_2=0.2$, where the system is near to line of phase transition in the graphic $D_C$ vs. $J_2$: $0.251\le J_{2C}\le 0.252$, where $D_C$ goes continuously to zero $D_C\rightarrow 0$^33^. There is a discontinuous drop of $D_C$ at $J_{2C}$ which is an effect of the dimension of the system. Being the behavior for three-dimensional and two-dimensional model are similar as was found in Ref.^32^. Where below $D_C$ the system is ordered and the magnetization is non-zero and the magnetization goes to zero at $D_C$. We obtain a large peak for the spin conductivity at $\omega \approx 0.03$ and a finite spin conductivity at $\omega \rightarrow 0$ and none divergence at DC limit, which is due to the behavior of the expression for $\sigma ^{reg}(\omega )$. Furthermore, this peak is due to behavior of dispersion relation $\omega _\mathbf{k }$ in the Brillouin zone, where the main effect of the frustration occurs at the long-wavelength limit where the gap of the dispersion relation, in the disordered phase vanishes at critical parameter $D_C\rightarrow 0$, at a wave vector signaling the magnetic order that appear below $D_C$. Experimental results can be compared with our results when available. As far as I know, there is no result for the spin transport for the model studied here. As In Fig. 5, we obtain $\sigma ^{reg}(\omega )$ as a function of $J_2$ and for the values: $J_1=-1.0$ and $J_y=0.1$. We perform the calculations for the $\omega$ near to the peak of the spin conductivity obtained in Fig. 4, $\omega _0=0.03$. Due to symmetry of equation for conductivity, we should obtain the same behavior for the conductivity as a function of $J_y$. In Fig. 6, we obtain $\sigma ^{reg}(\omega )$ as a function of $J_2$ for $J_1=-1.0$ and $J_y=0.1$. We perform the calculations also near to the peak of the spin conductivity, $\omega \approx \omega _0=0.03$. In this case, we obtain a monotonically increasing behavior for the conductivity as expected.

## Conclusions

In brief, we analyze the effect of scattering among electrons with a ferromagnetic wall domain on spin transport by electrons and analyze the spin transport by quasi particles in the frustrated Heisenberg ferromagnet with in-chain and inter-chain interactions which is very important in the field of antiferromagnetic and ferromagnetic spintronics. Additionaly, the Meissner effect for the spin superconductivity obtained for many other frustrated spin systems is also proposed. We analyze the case of a lattice model described by the isotropic Heisenberg model with a ferromagnetic in-chain interaction $J_1<0$ between nearest neighbors and an antiferromagnetic next-nearest-neighbor in-chain coupling $J_2>0$. We obtain a large variation of the spin conductivity with the frustration parameters: $J_2$, $J_{\perp ,y}$ and $J_{\perp ,z}$. There is also a Drude weight for the spin conductivity which exists in many other spin models. However, the purpose here is to analyze the effect of phase transition on continuum conductivity $\omega \ne 0$ where the Drude weight term does not generate any influence. The peak of the spin conductivity can be determined by measuring of magnetization current^44,50^. In Ref.^51^, some experimental techniques were proposed and seem to be feasible. Another experimental technique that can be used is the nuclear magnetic relaxation (NMR). The spin transport in the compound AgVP$_2$S$_6$ was experimentally investigated using this technique (NMR) in Ref.^52^, where the experiment was performed at high temperatures where the behavior for the spin transport is diffusive.

## Appendix: SU(3) Schwinger bosons

We consider the model given by

45 $$\begin{aligned} \mathcal {H}=J_1\sum _{\langle i,j\rangle ,x}S_i\cdot S_j+J_2\sum _{\left[ i,j\right] ,x}S_i\cdot S_j+J_{\bot ,y}\sum _{\langle i,j\rangle ,y}S_i\cdot S_j +J_{\bot ,z}\sum _{\langle i,j\rangle ,y}S_i\cdot S_j+D\sum _{i}(S_i)^2. \end{aligned}$$

The SU(3) Schwinger boson theory is a theory proposed in Refs.^53,54^ and is well adequate to study the anisotropic XXZ model with single-ion anisotropy *D* in the range $D>D_c$, for $N\rightarrow \infty$ limit where three boson operators $t_x$, $t_y$ and $t_z$ are defined as

46 $$\begin{aligned} t_x^{\dag }|v\rangle =|x\rangle , t_y^{\dag }|v\rangle =|y\rangle , t_z^{\dag }|v\rangle =|z\rangle , \end{aligned}$$

where $|v\rangle$ is the vacuum state. In terms of these $t_{\gamma }$ operators ($\gamma =x,y,z$), we write the spin-*S* operators as

47 $$\begin{aligned} S^{x} = -i(t_{y}^{\dag }t_{z}-t_{z}^{\dag }t_{y}), S^{y} = -i(t_{z}^{\dag }t_{x}-t_{x}^{\dag }t_{z}), S^{z} = -i(t_{x}^{\dag }t_{y}-t_{y}^{\dag }t_{x}). \end{aligned}$$

We introduces other boson operators $u^{\dag }$ and $d^{\dag }$ given by

48 $$\begin{aligned} u^{\dag }=\frac{1}{\sqrt{2}}(t_{x}^{\dag }+it_{y}),\,d^{\dag }=\frac{1}{\sqrt{2}}(t_{x}^{\dag }-it_{y}). \end{aligned}$$

Thus

49 $$\begin{aligned} |1\rangle =u^{\dag }|v\rangle ,\,|0\rangle =t_{z}^{\dag }|v\rangle ,\,|-1\rangle =d^{\dag }|v\rangle , \end{aligned}$$

In addition, we impose the constraint condition $u^{\dag }u+d^{\dag }d+t_{z}^{\dag }t_{z}=1$. We employ the further condition that $\langle t_z\rangle =t$^54^ and introduce the Lagrange multiplier $\mu _j(T)$ that is a chemical potential of bosons depending on each site *j* of the lattice and temperature. From the mean-field approximation, we let $\mu _j(T)=\mu (T)$. The other parameters in theory: $t^2$, $\mu$, $p_1$, $p_2$, $p_y$ and $p_z$ are obtained numerically by a set of integral equations as following

50 $$\begin{aligned} 2-t^2=  \frac{1}{N}\sum _{{{\mathbf {k}}}}\frac{\Lambda _{{\mathbf {k}}}}{\omega _{{\mathbf {k}}}}\coth \left( \frac{\beta \omega _{{\mathbf {k}}}}{2}\right) , \end{aligned}$$

51 $$\begin{aligned} \mu=  \frac{1}{N}\sum _{{{\mathbf {k}}}}\frac{\Lambda _{{\mathbf {k}}} -\Delta _{{\mathbf {k}}}}{\omega _{{\mathbf {k}}}}g_{{\mathbf {k}}} \coth \left( \frac{\beta \omega _{{\mathbf {k}}}}{2}\right) , \end{aligned}$$

52 $$\begin{aligned} p_1=  -\frac{1}{N}\sum _{{{\mathbf {k}}}}\frac{\Lambda _k}{\omega _k} \cos k_x\coth \left( \frac{\beta \omega _k}{2}\right) , \end{aligned}$$

53 $$\begin{aligned} p_2=  -\frac{1}{2N}\sum _{{{\mathbf {k}}}}\frac{\Lambda _{{\mathbf {k}}}}{\omega _{{\mathbf {k}}}}\cos 2k_x\coth \left( \frac{\beta \omega _{{\mathbf {k}}}}{2}\right) , \end{aligned}$$

54 $$\begin{aligned} p_y=  -\frac{1}{N}\sum _{{{\mathbf {k}}}}\frac{\Lambda _{{\mathbf {k}}}}{\omega _{{\mathbf {k}}}}\cos k_y\coth \left( \frac{\beta \omega _{{\mathbf {k}}}}{2}\right) , \end{aligned}$$

55 $$\begin{aligned} p_z=  -\frac{1}{2N}\sum _{{{\mathbf {k}}}}\frac{\Lambda _{{\mathbf {k}}}}{\omega _{{\mathbf {k}}}}\cos k_z\coth \left( \frac{\beta \omega _{{\mathbf {k}}}}{2}\right) . \end{aligned}$$

After performing a Fourier transformation followed by the Bogoliubov transformation

56 $$\begin{aligned} u_{{\mathbf {k}}}=\chi _{{\mathbf {k}}}\alpha _{{\mathbf {k}}}-\rho _{{\mathbf {k}}} \beta _{{\mathbf {k}}}^{\dag }, u_{{\mathbf {k}}} =\chi _{{\mathbf {k}}}\beta _{-{{\mathbf {k}}}}-\rho _{{\mathbf {k}}}\alpha _{-{{\mathbf {k}}}}^{\dag }, \end{aligned}$$

with

57 $$\begin{aligned} \chi _{{\mathbf {k}}}=\sqrt{\frac{\Lambda _{{\mathbf {k}}}+\omega _{{\mathbf {k}}}}{2\omega _{{\mathbf {k}}}}}, \rho _{{\mathbf {k}}} =\sqrt{\frac{\Lambda _{{\mathbf {k}}}-\omega _{{\mathbf {k}}}}{2\omega _{{\mathbf {k}}}}} \end{aligned}$$

we find

58 $$\begin{aligned} \mathcal {H}=\sum _{{{\mathbf {k}}}}\left( \alpha _{{\mathbf {k}}}^{\dag } \alpha _{{\mathbf {k}}}+\beta _{{\mathbf {k}}}^{\dag }\beta _{{\mathbf {k}}}\right) +\sum _{{{\mathbf {k}}}}\left( \omega _{{\mathbf {k}}}-\Lambda _{{\mathbf {k}}}\right) +C \end{aligned}$$

with

59 $$\begin{aligned}C=\mu N(1-t^2)-\frac{N}{2}(1-t^2)(J_1+J_2+J_y+J_z) +2N\left( J_1p_1^2+J_2p_2^2+J_yp_y^2+J_zp_z^2\right) \end{aligned}$$

60 $$\begin{aligned}\Lambda _{{\mathbf {k}}}=r+2t^2g_{{\mathbf {k}}}, \end{aligned}$$

61 $$\begin{aligned}r=(J_1+J_2+J_y+J_z)(1-t^2)-\mu +D \end{aligned}$$

62 $$\begin{aligned}g_{{\mathbf {k}}}= J_1\cos k_x+J_2\cos 2k_x +J_y\cos k_y+J_z\cos k_z \end{aligned}$$

63 $$\begin{aligned}\Delta _{{\mathbf {k}}}= 2J_1(t^2-p_1)\cos k_x +2J_2(t^2-p_2) \cos 2k_x+2J_y(t^2-p_y)\cos k_y+2J_z(t^2-p_z)\cos k_z, \end{aligned}$$

and $\omega _{{\mathbf {k}}}$ given by

64 $$\begin{aligned} \omega _{{\mathbf {k}}}=\sqrt{\Lambda _{{\mathbf {k}}}^{2}-\Delta _{{\mathbf {k}}}^{2}}. \end{aligned}$$

that are the dispersion relation for spin waves. Furthermore, the system present a gap in the spectrum that closes at $\mathbf {k}\in (\pi ,\pi )$.
